# Estimating Speed Error of Commercial Radar Tracking to Inform Whale–Ship Strike Mitigation Efforts

**DOI:** 10.3390/s25061676

**Published:** 2025-03-08

**Authors:** Samantha Cope King, Brendan Tougher, Virgil Zetterlind

**Affiliations:** ProtectedSeas, Anthropocene Institute, 2475 Hanover St. Ste 100, Palo Alto, CA 94304, USA; brendan@anthinst.org (B.T.); virgil@protectedseas.net (V.Z.)

**Keywords:** marine radar, target tracking, automatic identification system (AIS), vessel speed, vessel speed reduction, ship strikes

## Abstract

Vessel speed reduction measures are a management tool used to reduce the risk of whale–ship strikes and mitigate their impacts. Large ships and other commercial vessels are required to publicly share tracking information, including their speed, via the Automatic Identification System (AIS), which is commonly used to evaluate compliance with these measures. However, smaller vessels are not required to carry AIS and therefore are not as easily monitored. Commercial off-the-shelf marine radar is a practical solution for independently tracking these vessels, although commercial target tracking is typically a black-box process, and the accuracy of reported speed is not available in manufacturer specifications. We conducted a large-scale measurement campaign to estimate radar-reported speed error by comparing concurrent radar- and AIS-reported values. Across 3097 unique vessel tracks from ten locations, there was strong correlation between radar and AIS speed, and radar values were within 1.8 knots of AIS values 95% of the time. Smaller vessels made up a large share of the analyzed tracks, and there was no significant difference in error compared to larger vessels. The results provide error bounds around radar-reported speeds that can be applied to vessels of all sizes, which can inform vessel-speed-monitoring efforts using radar.

## 1. Introduction

Monitoring vessel activity is essential for understanding and managing risks posed to marine species by human activities on the water. The International Convention on Safety of Life at Sea (SOLAS) established operational standards for ships and currently mandates the use of the Automatic Identification System (AIS), requiring that ships broadcast tracking information [1]. This includes static information, such as identification and size, and dynamic information, such as vessel position and speed throughout a voyage (see [2] for a full list of information provided). AIS data have been used widely to manage vessel activity and inform environmental impacts [3,4]. But the SOLAS standards do not apply to smaller vessels, like pleasure craft and fishing vessels [1]. Additional monitoring tools are necessary to incorporate these vessels into similar management strategies.

Vessel speed reduction measures have been deployed in coastal areas where vessel activity overlaps species’ habitat to reduce the risk of whale–ship strikes [5,6]. Depending on the measure, vessels are required or requested to adhere to speed limits. AIS is commonly used to evaluate adherence with these measures (e.g., [7,8,9,10,11]). But smaller, non-SOLAS vessels are also required to obey the limits in some areas. For example, in Canada and the USA at present, vessels of at least 13 m and 20 m, respectively, must obey seasonal speed limits established to prevent collision with the endangered North Atlantic right whale population [12,13]. On the U.S. east coast, pleasure craft have the lowest level of compliance with this speed measure, and there have been known injuries and mortalities from strikes with these vessels [9].

Recent research efforts have revealed that a significant amount of smaller vessel traffic does not use AIS, especially in coastal areas where whale habitat and human activity overlap [14,15,16] and rates of non-compliance with existing management measures are greatest [9,17]. Therefore, vessel strike risk will likely be underestimated in coastal areas if vessels without AIS are ignored [18]. Further, smaller vessels that are less likely to use AIS can travel at much higher speeds than larger commercial ships. AIS is a valuable source of vessel data due to global use and well-documented standards, but other methods for monitoring vessel speed and activity are emerging to fill critical gaps in data on small vessel activity in whale habitat.

The original SOLAS standards identified radar as a critical component for vessel operators to monitor the location of other vessels on the water [19]. X-band (8–12 GHz) marine radar is also commonly used to manage vessel traffic in busy port areas [20]. By measuring the distance and bearing to vessels (i.e., the position relative to the radar), marine radar systems provide their location on the water. A vessel’s speed and course (its direction of travel) have been determined traditionally by measuring the change in these positions over time. Modern systems automate this process, called target tracking, or also often referred to as the Automatic Radar Plotting Aid (ARPA).

Today, marine radar is a widely used and available sensor system for boaters. In the same way that radar supports safe navigation and vessel traffic systems, radar is a valuable method for independently monitoring smaller vessels, since they can be detected without the need for participation in AIS or other systems. Coastal radar has been used previously to track and incorporate small vessels into underwater noise modeling [21], to evaluate trends in boater behavior and small-scale fisheries in marine protected areas [22,23], and to integrate small vessels with larger-scale surveillance of coastal areas [24]. Therefore, radar has strong potential for application to vessel monitoring in support of whale–ship strike mitigation.

The primary objective of this study was evaluating error in vessel speeds reported by commercial radar tracking to support the monitoring and enforcement of speed reduction measures for all vessels but especially for smaller, non-SOLAS vessels. Error in vessel course was also included, as it is another component of target tracking and may inform other monitoring efforts. Radar is an established tool for measuring vehicle speed on roadways, albeit with differences in system design [25]. When used to enforce speed limits, its value as evidence against offenders often depends on its accuracy [26]. At the time of writing, the main recommendation of performance standards for marine radar equipment, Ref. [27], defines the required speed and course accuracy of radar systems used by ships complying with SOLAS, but these standards are only required to be met during unspecified testing scenarios, and accuracy can be reduced in real-world situations [28].

Radars used by SOLAS vessels are advanced systems governed by international standards and are much higher cost than commercial off-the-shelf (COTS) radar systems purchased and used by boaters. The lower cost of COTS radars makes this sensor system more applicable and accessible for conservation and research efforts. But without the most advanced hardware and software, these radars are not SOLAS-compliant and therefore not required to meet the SOLAS standards. Further, published manufacturer specifications for these products do not widely share proprietary information on target tracking, making this a largely black-box process. Therefore, there is a need to understand and quantify the typical and realistic error that can be expected to support monitoring efforts where lower-cost COTS radar is a more appropriate tool.

Without published specifications or detailed proprietary information available, this study reports the results and statistical summary of a large-scale measurement campaign to evaluate the speed and course error of commercial radar tracking. We utilized an in situ, empirical approach to establish error bounds across a large sample of field measurements from multiple locations. AIS data were used as “truth” to evaluate absolute accuracy, similar to previous work (discussed further in Section Related Work). This study significantly expands the sample size and is the first of its kind to include data from Class B AIS, an optional version of AIS for smaller, non-SOLAS vessels. Incorporating a variety of vessel types also supported a statistical analysis to investigate whether speed and course error are related to vessel size or other variables. Ultimately, results provide a systematic, widely replicated evaluation of accuracy for all sized vessels in real conditions. COTS radar users will be able to apply the error bounds to the reported speeds to more confidently assess compliance where speed reduction measures are in place or are being considered.

The following sections are structured as follows: Section Related Work provides a summary of published work developing target-tracking algorithms for marine radar and other approaches for using radar to monitor vessel speed. Section 2.1 describes the radar and AIS data collection methods, including known information about the proprietary target-tracking algorithms. Section 2.2 provides details on the analysis methodology, including associating radar and AIS data and evaluating the error across data sources and vessel characteristics. The results are reported in Section 3, and Section 4 compares the estimated error to other studies, identifies potential sources of error, and discusses how the results can be interpreted for monitoring both large and small vessels.

### Related Work

Despite the limited details available on the specific algorithms used by COTS radar systems, there has been extensive work published on target tracking for maritime radars, most commonly the Kalman filter algorithm or modifications of this approach [29,30]. Kalman filtering techniques track objects in linear motion by continuously predicting the state of a target (i.e., position and movement vector) based on the prediction error of former states [31]. Filtering is especially useful for tracking vessels against sea-clutter with X-band radar (e.g., [32,33,34]) and high-frequency radar (e.g., [35,36]). But since the predicted state is based on previous states with the Kalman filter, target maneuvers can diminish tracking accuracy.

Variations of the Kalman filter have been implemented to account for non-linear trajectories, including the common Extended Kalman Filter [37] and Unscented Kalman Filter [38]. These traditional methods for target tracking have been adapted in recent years for unmanned surface vehicles so they can avoid obstructions as they autonomously navigate on the water [39,40,41,42]. Other modern models have improved tracking performance using deep learning [43,44,45] and by incorporating features beyond a target’s movement information, such as target shape, (e.g., [46,47]), although these multi-step methods may not be suitable for real-time application across varied marine environments and vessel types.

The reliability of a target-tracking system largely depends on the accuracy of the tracking filter used. For ships complying with SOLAS, Ref. [27] does not stipulate a specific filtering method but does define some of the accuracy requirements. The positional error of marine radar tracking has been well studied (e.g., [33,48,49,50,51,52]). Experiments commonly employ a comparison of radar-reported data with data provided by AIS to assess radar accuracy. AIS uses onboard global navigation satellite system (GNSS) receivers (i.e., GPS or similar satellite-positioning systems) to report geolocation over time, a highly accurate method for identifying speed [53,54,55] as opposed to measuring it remotely via radar. Associating radar and AIS data is typically carried out by either interpolating AIS data points to synchronize with existing radar points [33,50] or pairing distinct data points from each based on similar characteristics [30]. AIS data have also been used to inform and evaluate new developments in target tracking (e.g., [33,34]).

A smaller number of studies have focused more specifically on the speed or course accuracy of target-tracking output, primarily as it relates to navigational safety for mariners. Perhaps the most extensive in situ work has been [56], which used SOLAS-compliant S-band and X-band radar systems onboard three ships to track 55 unique vessels. Simultaneous radar tracking and AIS data were used to estimate radar-reported speed and course error and ultimately compared to the corresponding SOLAS standards. Most vessels included in the analysis were larger ships, although four vessels were less than 100 m in length. Using a shore-based X-band radar, Ref. [33] evaluated the accuracy of a proposed tracking algorithm by comparing output speeds to AIS data from five unique vessels. Simulations using SOLAS-compliant radar systems by [57,58] have demonstrated that speed and course standards are not achievable in all tracking scenarios.

It is also important to note that satellite-borne radar is also used to look at vessel presence across large areas (e.g., [59,60]); however, the temporal lag of detections and infrequent update rate can limit real-time enforcement applications [61]. This is especially relevant when reconstructing vessel trajectories and estimating speed from subsequent detections, although some recent work has been able to relate the appearance of vessel wake in satellite imagery with vessel speed [62,63]. Given these constraints, traditional marine radar systems still offer benefits for active vessel management, including widespread availability and use.

## 2. Materials and Methods

**Figure 1:**
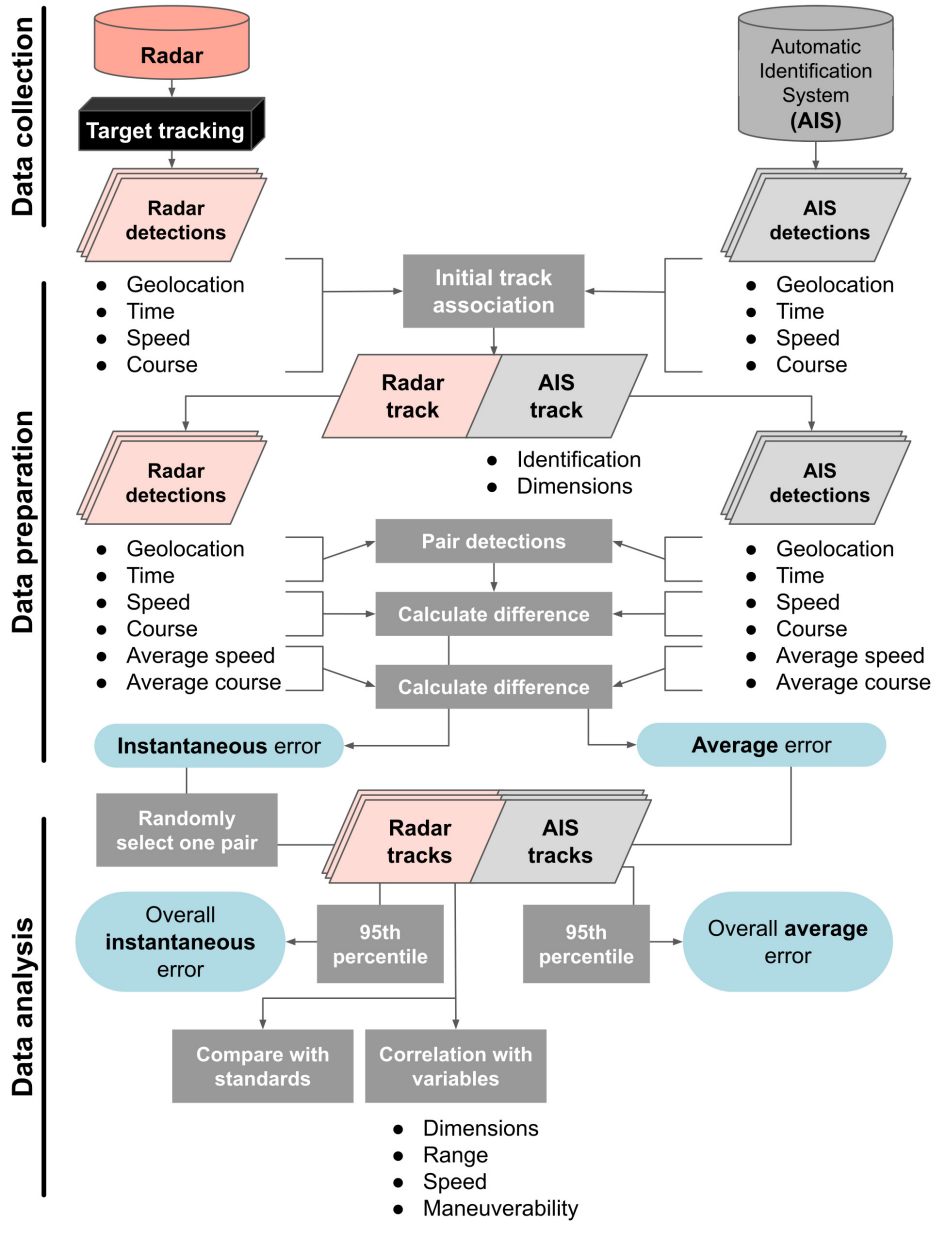
Conceptual diagram of data collection, preparation, and analysis methods.

An overview of the data collection, preparation, and analysis methods is shown in Figure 1. Initially, tracking information was collected simultaneously from both radar and AIS and used to associate records from both sensors that represented the same unique vessel’s trajectory. Then, individual radar and AIS data points were paired, and speed and course error were calculated as the difference between radar- and AIS-reported values. The 95th percentile of error across all vessel records was calculated as the overall error. Finally, instantaneous error was compared with SOLAS standards and other variables related to vessel size and activity characteristics.

### 2.1. Data Collection

All data were collected using shore-based Marine Monitor (M2) systems, a multi-sensor platform for independently monitoring vessel activity in coastal areas. These systems primarily utilize X-band marine radar systems to detect and track vessels on the water but also integrate additional components, including an AIS receiver. Software captures and stores incoming data from both (see [64] for full description). M2 systems are typically positioned along the coast to monitor specific nearshore managed areas, such as protected areas or port access, so a variety of vessel types are observed, including commercial ships and recreational vessels. Data are actively used to identify compliance with regulations and inform trends in activity.

**Figure 2:**
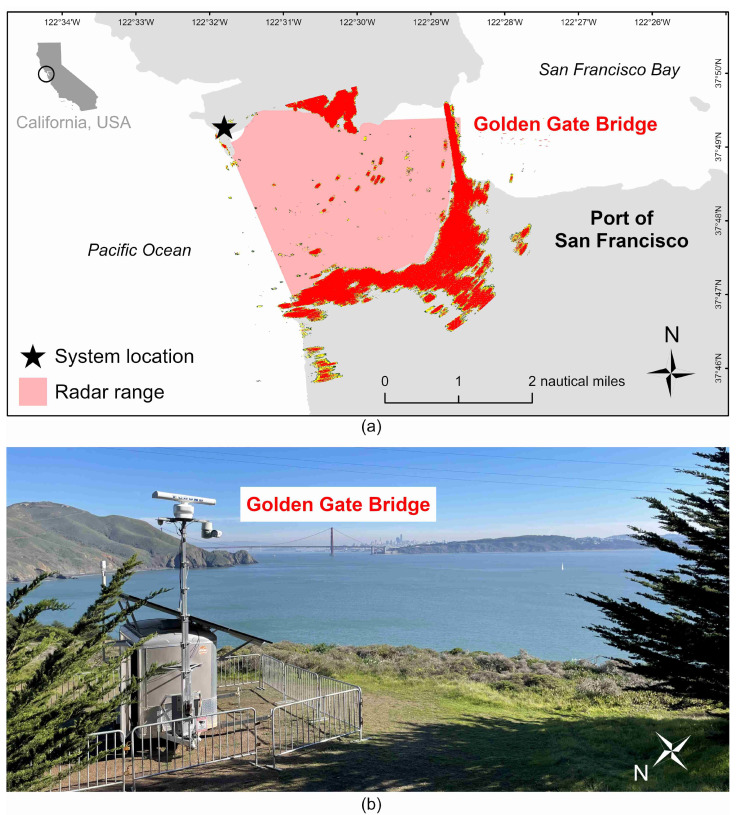
The Point Bonita location near San Francisco, California, USA including example radar imagery (**a**) and the point of view from the system (**b**). The location of the Golden Gate Bridge is noted in both (**a**,**b**) for reference.

This study utilized data collected at ten M2 system locations across the USA and Mexico. Some systems were located in the same region, but each monitored a unique area, and there was no overlap in observed ranges. The configuration of one of these locations, Point Bonita near San Francisco, California, is shown in Figure 2. Other specific locations are not disclosed for safety and security purposes but are identified as Location A, B, etc. M2 systems typically operate continuously, but occasional disruptions in data collection occur during periods of system maintenance. For the data analyzed in this study, we opportunistically selected time periods from each location when systems were fully operational (Table 1).

#### 2.1.1. Radar

The radar antennas used at all ten locations were manufactured by Furuno with either magnetron or solid-state transmitters. Solid-state radars offer practical benefits over magnetron radars, like improved reliability and lower power requirements [28], and are capable of using the Doppler effect to estimate the radial velocity of targets. The specific model used at each location varied depending on the specific monitoring needs of the area. Details on the specifications of each antenna are shown in Table 2. These radars are intended for recreational and fishing applications (non-SOLAS) but do meet the same positional accuracy requirements of radars used on ships that comply with SOLAS [27].

The initial configuration for each location included deploying the radar antenna and mast on level ground, identifying the geolocation of the radar antenna, and setting the radar heading alignment referenced to true north. Masts were constructed of steel or aluminum, depending on availability, and leveled using outriggers if not initially on a level surface. We identified the antenna location using GPS and set the fixed location in the radar system software, which requires the degrees decimal minutes format with seconds accurate to three decimal places (e.g., 37°49.272′ N, 122°31.777′ W), using the ground-stabilized mode. For the radar heading alignment, we identified the difference in true north to the facing of the radar antenna and set this offset in the radar system software accurate to one degree. To ensure that it was accurately oriented, we manually checked that the position of known objects such as land, channel markers, and other structures were correctly identified and made any necessary adjustments to the heading offset value in the software.

Radar settings were configured to optimize tracking at each unique location, crucial for ensuring vessels of interest are successfully detected and minimizing the impact of location-specific environmental conditions [28]. The range scale of the radar, which determines the area over which the system can detect targets, was set so that vessels of any size were likely to be detected within 5 nautical miles (9.26 km). Adjustments were made if there were targeted areas of concern very close to shore or farther than 5 nautical miles. Radar controls for minimizing the impact of surface conditions and rain on target tracking were automatically set by the radar processor, except when this functionality did not effectively adapt to highly variable conditions. Finally, settings to identify minimum and maximum sizes of tracked targets were adjusted based on the size of vessels typically active in the area.

Depending on the location, target tracking was provided by the Furuno radar directly or Nobeltec TimeZero Coastal Monitoring navigation software. TimeZero Coastal Monitoring software uses an advanced Kalman filter for target tracking and outputs an update once per second per unique target [66], although further details are not published. Furuno does not specify the type of algorithm used [65]. M2 software archives new updates every 2 s to control data storage and the processing load on computers in the field (e.g., [67]). Target tracking at locations with solid-state radar incorporated the radial velocity of targets in tracking algorithms. All locations utilized the automatic acquisition functionality of target tracking, whereby the radar processor selects targets and tracks them over time. Radar systems (and AIS data) reported speed over ground (SOG) in knots and course over ground (COG) in degrees referenced to true north.

#### 2.1.2. AIS

A very-high-frequency (VHF) antenna and AIS receiver collected AIS messages from vessels in the area of M2 systems. In addition to the dynamic information provided, like SOG and COG, AIS data also contain static information, like vessel type and dimensions [2]. Data from Class B equipment used by some non-SOLAS vessels do not typically include this static information [3], but the dynamic information provided a means to include smaller vessels in analysis. Class B vessels were identified as such by the specific AIS sentence information for positional updates, which is unique to Class B and does not provide any static data other than their Maritime Mobile Service Identity (MMSI). To summarize vessel types included in analysis, we used the ship-type codes included in static AIS data [68].

Errors in both static and dynamic AIS data have been well documented [4,54,55,69]. AIS data from vessels are provided by onboard equipment which has the potential to report data incorrectly if malfunctioning or not properly configured by the vessel operator. However, rigorous certification is required for both Class A AIS [70] and Class B AIS [71,72] equipment manufacturers. Further, GNSS used by AIS on SOLAS vessels are required to meet SOG and COG reporting accuracy standards under normal operating conditions (e.g., [73]) (discussed further in Section 4). Previous research has demonstrated that using large data sets minimizes the impact of occasional errors in AIS data on overall results [8,74].

### 2.2. Data Preparation

#### 2.2.1. Radar and AIS Track Association

Both the radar system and AIS provided target positions over time, along with estimated SOG and COG, hereafter referred to as detections. Consecutive detections of unique radar targets were linked using a common identification number, hereafter referred to as tracks. AIS detections from unique vessels were linked similarly. The time interval between AIS broadcasts varies based on speed, with slower vessels generally broadcasting data less frequently (e.g., every 10 s when traveling less than 14 knots), and Class B less frequently overall [2]. The interval at which detections were recorded from the radar systems was typically between 2 and 8 s. When a vessel tracked by radar was also broadcasting AIS data, there were two sets of records for the same vessel, and coincident radar and AIS detections were used to initially associate the two track records of the same vessel (see Table 3 for a sample of detection data). Coincident detections were associated according to requirements recommended by [30], including similar position, time, speed, and course (detailed in [64]):

- detections are within 100 m in geolocation;
- detected within 15 s;
- difference in SOG of less than 1.5 knots (0.77 m/s); and
- difference in COG of less than 10 degrees.

Detections and tracks were stored and associated within an SQL database at the time data were received and were later extracted for further analysis in R [75].

All track records collected by M2 systems during the periods of data collection were initially filtered to select only those associated radar and AIS tracks that provided sufficient and non-erroneous data from both sources: tracks that lasted longer than 5 min and vessels with reported speeds below 50 knots. In the case that more than one radar track was associated with a single AIS track, the radar track with more associated points was selected. Given that the range of AIS was typically larger than the range at which the radar systems were configured [28], the portions of AIS tracks that extended beyond the radar range, and thus not possibly associated with radar detections, were ignored.

**Table 3:** A sample of consecutive detections of a cargo vessel at the Point Bonita location. All detections represent the same vessel with radar detections in red and detections from the Automatic Identification System (AIS) in gray. Position, time, speed over ground (SOG), and course over ground (COG) variables were used to associate radar and AIS detections. Associated detection pairs are identified A–E.

Track Number	Latitude (Decimal Degrees)	Longitude (Decimal Degrees)	Time	SOG (Knots)	COG (Degrees)
26810252	37.815915	−122.493363	18:56:32	8.7	254.6
26810252	37.815835	−122.493798	18:56:40	9.0	255.1
26810252 ^A^	37.815743	−122.494220	18:56:48	8.9	255.2
26809029 ^A^	37.815792	−122.494208	18:56:54	9.0	254.0
26810252 ^B^	37.815673	−122.494627	18:56:56	8.9	255.8
26809029 ^B^	37.815677	−122.494722	18:57:03	9.1	254.2
26810252	37.815605	−122.495022	18:57:05	8.8	256.2
26810252	37.815495	−122.495427	18:57:13	8.9	254.8
26810252 ^C^	37.815392	−122.495817	18:57:20	8.9	253.9
26809029 ^C^	37.815433	−122.495723	18:57:23	9.1	253.1
26810252 ^D^	37.815267	−122.496213	18:57:28	9.0	252.2
26809029 ^D^	37.815288	−122.496225	18:57:34	9.1	250.8
26810252 ^E^	37.815185	−122.496563	18:57:36	8.6	252.8
26809029 ^E^	37.815142	−122.496672	18:57:43	9.0	249.1

#### 2.2.2. Radar and AIS Detection Pairing

For this study, all detections along associated tracks were re-evaluated to identify associated pairs but only considering position and time (similar to [49]). In this way, the SOG and COG criteria used to initially associate the track records did not limit the maximum SOG or COG error observed across data sources. We chose to pair existing detections (instead of interpolating AIS data to synchronize with radar detections) so that observed values would be used directly (discussed further in Section 4).

**Figure 3:**
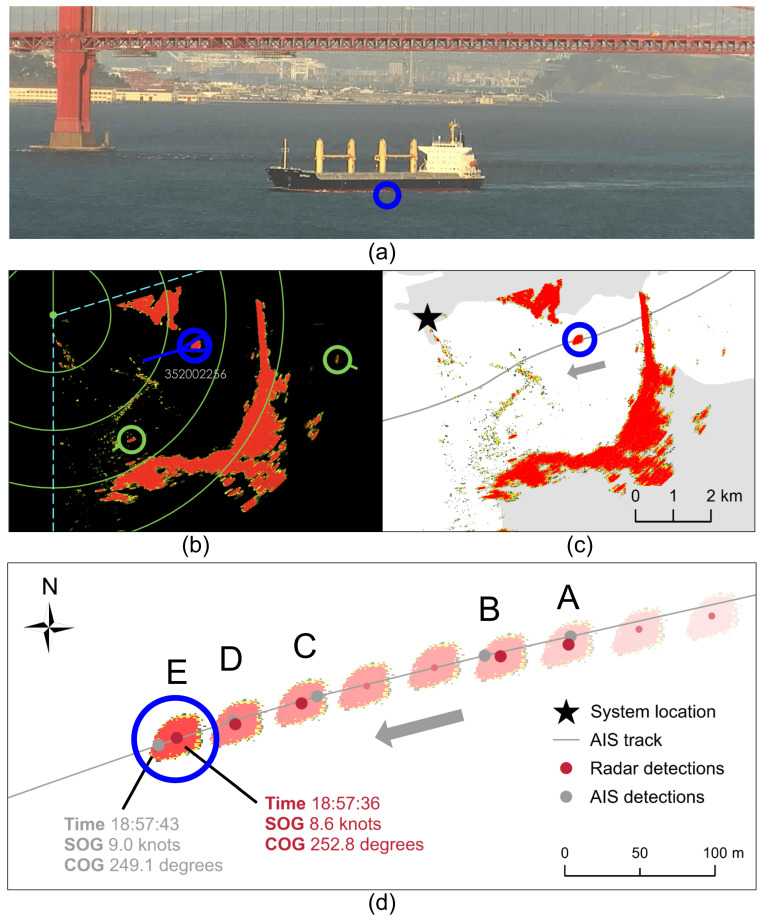
Cargo vessel transits the Point Bonita location (**a**) tracked by radar and the Automatic Identification System (AIS) (**b**,**c**). Associated detection pairs (**d**) are identified A–E and correspond with those in Table 3. Time, speed over ground (SOG), and course over ground (COG) are noted for detection pair E as an example. The AIS track line connects consecutive AIS detections and is shown for reference.

Each unique AIS detection was paired with the unique radar detection closest in distance (see examples in Figure 3). Pairs were discarded from analysis if the distance between them was greater than the radar’s range accuracy (1% of the range scale in use) [28] as this could indicate an error in tracking. Analysis was limited to pairs with a time difference of less than 15 s between them (similar to the initial association criteria). Given the frequency of radar detections (roughly every 2 and 8 s), a time window of 15 s provides an acceptable resolution to capture associated detections. Pairs were also limited to those that occurred without the vessel abruptly maneuvering, defined as a change in SOG greater than 25% or change in COG greater than 15 degrees [76]. This aimed to prevent pairing a detection that occurred prior to a maneuver with one that occurred after. Detections that occurred during the first minute of the radar track were ignored as accuracy is likely to be much lower while target-tracking algorithms process the initial detections [28]. Finally, if multiple radar detections were paired with a single unique AIS detection, the radar detection with the lowest difference in both distance and time was selected.

### 2.3. Data Analysis

#### 2.3.1. Error Calculations

Error in radar-reported SOG and COG was defined as the absolute difference between the values reported by the radar system and those provided in AIS data [77] across associated detection points and tracks. We calculated instantaneous error for detections (Equations (1) and (2)) and average error for tracks (Equations (3) and (4)). The average error can inform large-scale analysis; however, errors at unique points along a track provide an estimate of accuracy that can be expected at any given point of observation. This can be helpful when monitoring vessel activity in real-time. The ‘circular’ R package was used for circular COG calculations and statistical analysis [78]. Instantaneous error in SOG and COG was calculated at each associated radar and AIS detection pair (*x*) along a track according to Equations (1) and (2), respectively.

(1) $$SOG\;error_{x}=\left|\;SOG_{radar\;x}-SOG_{AIS\;x}\;\right|$$

and

(2) $$COG\;error_{x}=\left\{\begin{array}{c} |\;COG_{radar\;x}-COG_{AIS\;x}\;|,\;\;\;\;\;\;\;\;\;\;\;\;\mathrm{if}|\;COG_{radar\;x}-COG_{AIS\;x}\;|\leq 180{}^{\circ},\\ 360{}^{\circ}-|\;COG_{radar\;x}-COG_{AIS\;x}\;|,\;\;\;\mathrm{if}|\;COG_{radar\;x}-COG_{AIS\;x}\;|>180{}^{\circ}. \end{array}\right.$$

Average error in SOG and COG was calculated using Equations (1) and (2), but substituting the average SOG and COG of all detection points (*n*) along a track according to Equations (3) and (4), respectively.

(3) $${\bar{SOG\;error}}_{n}=\left|\;{\bar{SOG}}_{radar\;n}-{\bar{SOG}}_{AIS\;n}\;\right|$$

and

(4) $${\bar{COG\;error}}_{n}=\left\{\begin{array}{c} |\;{\bar{COG}}_{radar\;n}-{\bar{COG}}_{AIS\;n}\;|,\;\;\;\;\;\;\;\;\;\;\;\;\mathrm{if}|\;{\bar{COG}}_{radar\;x}-{\bar{COG}}_{AIS\;x}\;|\leq 180{}^{\circ}\\ 360{}^{\circ}-|\;{\bar{COG}}_{radar\;n}-{\bar{COG}}_{AIS\;n}\;|,\;\;\;\mathrm{if}|\;{\bar{COG}}_{radar\;x}-{\bar{COG}}_{AIS\;x}\;|>180{}^{\circ}. \end{array}\right.$$

For radar systems that are required to meet SOLAS standards, speed and course error must be below a defined threshold 95% of the time [27]. A similar statistical methodology was used to estimate overall error across all analyzed radar tracks. Each track was represented by a single instantaneous error value and its average error value. Most radar tracks had more than one detection associated with AIS detections (29 on average), so a single pair of detections was randomly selected from each radar track so that results were not biased by track duration. Overall instantaneous and average error were determined by calculating the 95th percentile of track instantaneous and average error values. Correlation is often used to evaluate the similarity of associated radar and AIS observations (e.g., [79,80,81,82]). SOG at the selected detections was normally distributed, so we selected the Pearson correlation coefficient (*r*) to evaluate the strength and significance of the relationship between radar- and AIS-reported values. For COG, we utilized a circular version of the Pearson correlation [83].

#### 2.3.2. Comparison with Standards

The SOLAS standards are not required to be met outside of testing scenarios; however, the error was compared to these threshold values for reference and for comparison with similar experiments. To satisfy the standards, reported speed and course error must be within 0.5 knots and 5 degrees, respectively, 95% of the time after three minutes of steady-state tracking [27], meaning that no significant maneuvers have occurred [28]. Therefore, the first detection pair to occur after the first three minutes of the radar track was selected. Similar to [56], the percentage of tracks meeting the standard after three minutes was calculated.

#### 2.3.3. Correlation with Variables

Finally, we explored the relationships between error and vessel size, range, speed, and maneuverability. Instantaneous SOG and COG error at selected detection points were compared to the associated AIS data. Small vessels have a smaller radar cross-sectional area (RCS) compared to larger vessels [27], and this reduced reflectivity can make smaller targets more difficult to detect and track [28]. Error associated with smaller vessels was a primary focus of this study, but radar tracking error of larger vessels can also occur due to misidentification of the target center when the vessel’s size exceeds the resolution of the radar system. The planar dimensions of vessels are provided in AIS, so vessel length was selected to represent vessel size. A vessel’s RCS varies depending on its aspect angle relative to the radar system (i.e., the RCS will only reflect the full vessel length as it passes across the radar’s line of sight at 90 degrees). Length calculations did not account for aspect given that most M2 locations have a similar orientation to Point Bonita (Figure 2), where vessels primarily transit past the radar. We also assume that the rate of change in RCS length with aspect is constant regardless of vessel size, although the impact on tracking is likely more significant for smaller vessels (discussed further in Section 4).

Static AIS data provide vessel dimensions relative to the position of its GPS antenna [68], so length overall was calculated according to Equation (5)

(5) length=a+b,

where *a* is the reported distance from antenna to bow, and *b* is the reported distance from antenna to stern (both in meters). Class B vessels did not report dimensions in AIS data, so they were grouped with the smallest vessels (those 20 m in length or shorter). International AIS requirements are based on the gross tonnage of ships, taking into account the nature of their voyages, whether international or domestic, with special provisions for passenger ships, regardless of size. The U.S. Coast Guard requires it for vessels 65 feet (roughly 20 m) and greater in length [84]. Vessels that reported a length of 0 were not included in the length analysis.

The distance to a vessel from the radar antenna is an important factor in radar tracking, given that the transmission frequency of marine radar limits the detection of targets to roughly line of sight [28]. If the vessel is below the horizon when viewed from the location of the radar, it will not be detectable. Thus, taller vessels should be visible at greater ranges compared to shorter vessels [27]. The theoretical detection range ($R_{d}$) (in nautical miles), including the effects of atmospheric conditions, can be calculated according to Equation (6) [85]

(6) $$R_{d}=2.21\left(\sqrt{h}+\sqrt{H}\right),$$

where *h* and *H* are the heights above sea level (in meters) of the antenna and the vessel, respectively. According to Equation (6), a small recreational vessel with 3 m air draft, such as a single-decked center console boat, would be detected up to roughly 16 km using a radar antenna 5 m above sea level, which was the typical height of the radar systems deployed onshore. Even within the detection range, the reduced RCS of smaller vessels may inhibit detection at greater distances. We identified the distance to vessels (i.e., range) by calculating the Euclidean distance between the site location and the latitude/longitude positions reported in AIS data.

Speed, course, and other related characteristics, such as turning rate, are important for representing a vessel’s trajectory [77] and ultimately identifying specific activities (e.g., fishing). Therefore, we selected two additional variables to compare with SOG and COG error: the SOG at which vessels traveled and their maneuverability, a measure of variation in COG. Maneuverability was calculated at each detection as the circular standard deviation of COG (*v*) across all AIS detection points over the prior kilometer, according to Equation (7) [86]

(7) $$v=\sqrt{-2\times \ln(\bar{R})},$$

where $\bar{R}$ is the mean resultant length of *n* vectors at angles $\theta_{n}$ calculated by Equations (8)–(11):

(8) $$\bar{R}=\frac{R}{n},$$

where

(9) $$R=\sqrt{C^{2}+S^{2}},$$

(10) $$C=\sum_{i=1}^{n}\cos\theta_{i},$$

and

(11) $$S=\sum_{i=1}^{n}\sin\theta_{i}.$$

To identify the strength and significance of the relationships between error and these variables, we selected the non-parametric Spearman rank-based correlation coefficient (ρ) as error was not normally distributed. Since the length of Class B vessels was unknown, we binned data so that Class B vessels were grouped with vessels 20 m in length or shorter. The remaining length values, and all range and SOG values, were grouped into bins of equal width to account for the full extent of observed values. Maneuverability values were highly skewed, so equal-frequency bins were selected using the 25th, 50th, and 75th percentile values. Since radar tracking is subject to errors from a host of interrelated variables [28], a full statistical investigation into the potential causes of error was beyond the scope of this analysis. We compared error with variables that are commonly used in vessel activity analysis to guide expectations of accuracy in future work.

## 3. Results

Across the ten locations, there were a total of 3097 unique radar tracks that were paired with AIS tracks at 89,853 associated detection points. A summary of tracks for the most common vessel types detected (representing 88% of all tracks used in analysis) is shown in Table 4. There were 1485 unique vessels represented in AIS tracks, identified using unique MMSI numbers, although some did not report an expected MMSI, e.g., 999999999. On average, associated detections were 7.77 km (±4.58 km standard deviation) (4.20 ± 2.47 nautical miles) from the radar system. The average distance between associated radar and AIS detections was 34.34 m (±24.63 m), and the average time difference was 7 s (±4 s).

**Figure 4:**
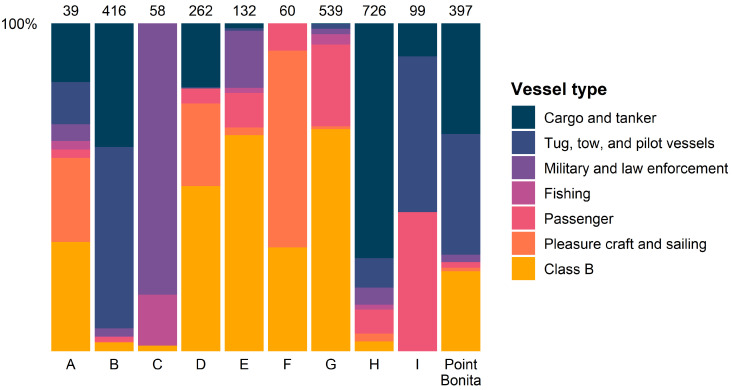
Vessel-type composition at each location for the most common vessel types detected. The total track counts are also noted for each location.

Each location had a unique composition of vessel types (Figure 4). Some locations were dominated by cargo and tanker ships, along with tug, tow, and pilot vessels that often assist them, including the Point Bonita location which reflects its proximity to major ports. Other locations were dominated by typically smaller vessels like passenger, pleasure craft, and Class B vessels.

**Figure 5:**
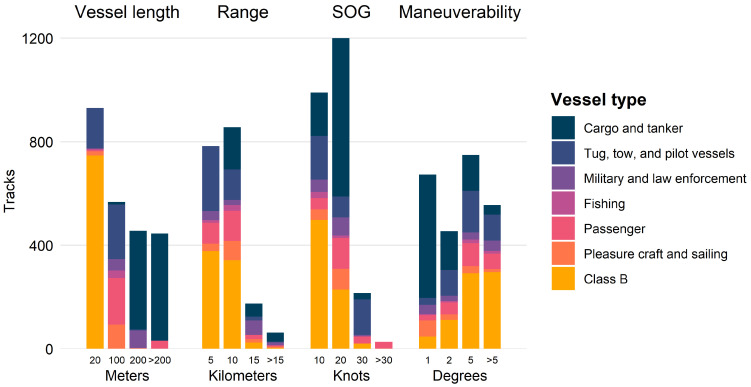
Total track counts by vessel length, range, speed over ground (SOG), and maneuverability for the most common vessel types detected. Values on the x-axis indicate the greater value of the intervals shown (i.e., data represented by 20 m in vessel length include records of vessels that were less than or equal to 20 m in length).

Most vessels were less than or equal to 100 m in length, and those that were greater than 100 m were primarily cargo and tanker ships (Figure 5). These larger ships primarily traveled at ranges greater than 5 km from the radar antenna. Smaller vessels, like pleasure craft and tug, tow, and pilot vessels, were most common within 10 km, likely the maximum detection range of the smallest vessels (discussed in Section 4). All of the most common vessel types traveled with SOG below 20 knots, with most vessels traveling between 10 and 20 knots. Vessels traveling faster than 30 knots were exclusively passenger vessels. Most vessels had low maneuverability. There were some vessels of all types that were associated with higher maneuverability, although most were smaller vessel types.

**Figure 6:**
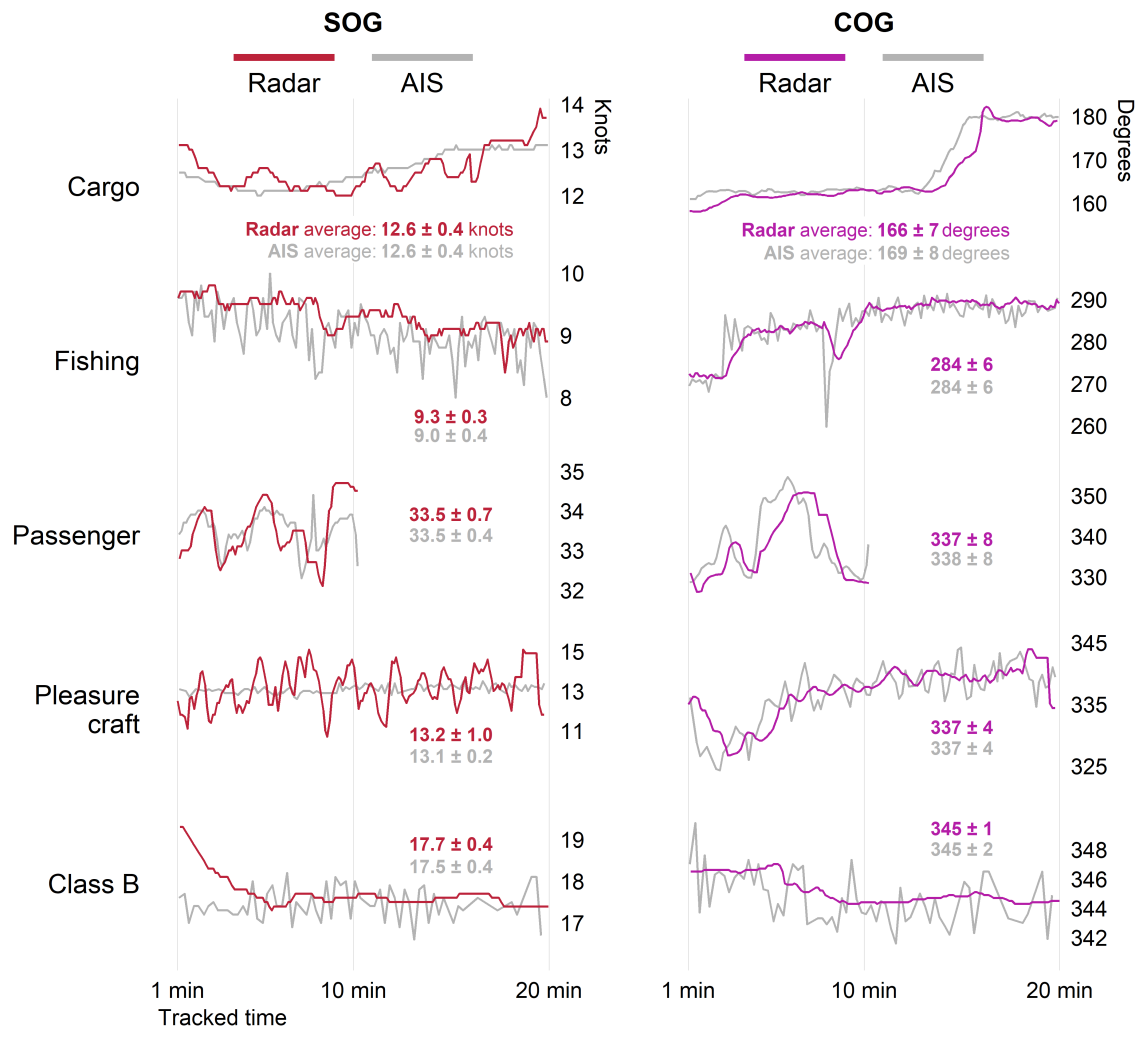
Instantaneous radar and AIS speed over ground (SOG) (**left**) and course over ground (COG) (**right**) for five example vessels. The average and standard deviation over the first twenty minutes of radar tracking are also shown.

Both the instantaneous and average SOG and COG from five example vessels are shown in Figure 6. These examples demonstrate radar-reported values that generally follow the patterns of SOG and COG over time reported by AIS, and average values from radar and AIS were similar.

### 3.1. Error

**Figure 7:**
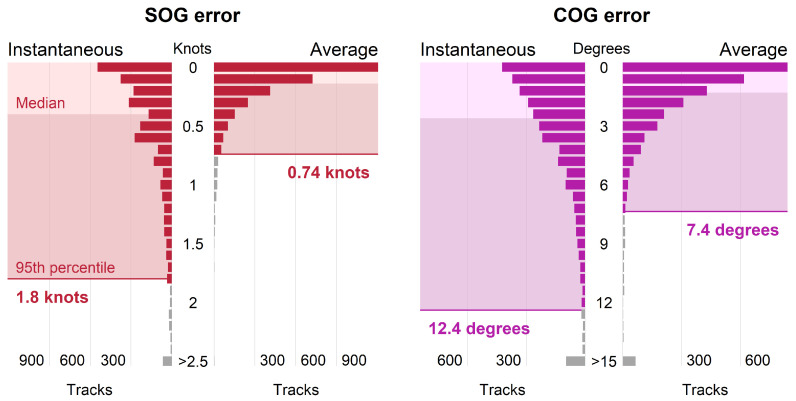
Distribution of radar tracks (*n* = 3097) by speed over ground (SOG) error (**left**) and course over ground (COG) error (**right**), including instantaneous and average error. The 95th percentile values are noted.

There was strong correlation between radar and AIS instantaneous SOG and COG (Pearson correlation coefficient (*r*) = 0.99, significance value (*p*) < 0.001, and degrees of freedom = 3095 for both variables). Both instantaneous and average error had skewed distributions, indicating that error was relatively low for most tracks (Figure 7), and average error was consistently lower than instantaneous error. The radar systems provided instantaneous and average SOG values within 1.8 knots (0.93 m/s) and 0.74 knots (0.38 m/s) of AIS-reported SOG, respectively, and instantaneous and average COG values within 12.4 and 7.4 degrees, respectively, 95% of the time, representing overall error.

**Figure 8:**
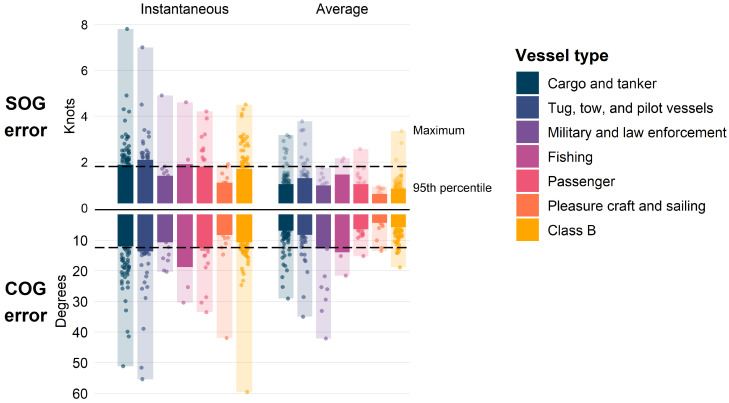
Speed over ground (SOG) and course over ground (COG) error for all selected points from the most common vessel types detected. Dashed line indicates overall instantaneous error.

Error varied by vessel type (Figure 8). Fishing vessels and tug, tow, and pilot vessels were associated with atypically high instantaneous error compared to the overall 95th percentile values. Military and law enforcement vessels, pleasure craft, and Class B vessels were associated with atypically low instantaneous error, although there were some outliers observed. The maximum instantaneous SOG and COG error was 7.8 knots and 59.6 degrees, respectively. Average error was generally much lower than instantaneous error for all vessel types except for the COG error of military and law enforcement vessels (discussed in Section 4).

### 3.2. Comparison with Standards

After three minutes of steady-state radar tracking, the instantaneous SOG error was within the SOLAS standard of 0.5 knots for 61% of radar tracks, and the COG error was within the SOLAS standard of 5 degrees for 74% of radar tracks.

### 3.3. Comparison with Variables

**Figure 9:**
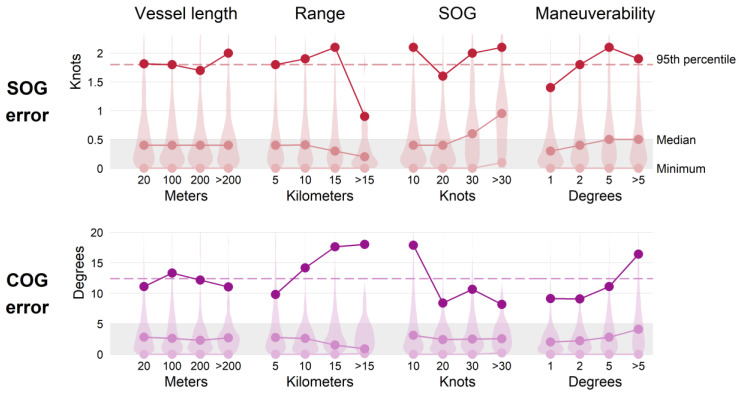
Instantaneous speed over ground (SOG) and course over ground (COG) error of radar tracks by vessel length (*n* = 2998), range, SOG, and maneuverability (*n* = 3097). Values on the x-axis indicate the greater value of the intervals shown (i.e., data represented by 20 m in vessel length include records of vessels that were less than or equal to 20 m in length). Red and purple shading indicate the relative distribution of error per group. Dashed line indicates overall instantaneous error. Gray shading indicates SOLAS accuracy standards for reference.

Error associated with vessel length, range, SOG, and maneuverability is shown in Figure 9. For each variable, the minimum, median, and 95th percentile error values of each data bin are notated as points and connected by lines to highlight any visible trends. There were no statistically significant relationships between instantaneous error and vessel length (Table 5). There was no correlation between SOG error and range, however there was weak negative correlation between COG error and range (Spearman correlation coefficient (ρ) = −0.064, significance value (*p*) < 0.001, and degrees of freedom = 3095). The median COG error decreased with greater range, although the associated 95th percentile increased which may indicate the presence of outliers at the largest ranges (discussed in Section 4). The distribution of data within each bin showed that the majority of tracks within each length and range bin met the SOLAS standards.

There was weak positive correlation between SOG error and SOG (Spearman correlation coefficient (ρ) = 0.058, significance value (*p*) < 0.01, and degrees of freedom = 3095). The median SOG error of the fastest vessels was more than twice the error of the slowest vessels (0.95 knots compared to 0.4 knots), although the 95th percentile value of these two groups was the same (2.1 knots). The strongest observed correlation between variables was that of COG error and maneuverability (Spearman correlation coefficient (ρ) = 0.255, significance value (*p*) < 0.001, and degrees of freedom = 3095) with a visible positive trend of increasing error with increasing maneuverability in both median and 95th percentile values (Figure 9).

## 4. Discussion

Results provide a reliable estimate of radar-reported SOG and COG accuracy by utilizing a large sample size. Previous research estimating error used much smaller sample sizes and limited vessel types but produced similar results. For example, across 55 unique vessels, Ref. [56] found that radar-reported SOG and COG accuracy was within SOLAS standards 49% and 69% of the time, respectively (compared to 61% and 74% in results presented here). The results also showed similar instantaneous speed error to X-band radar devices used for measuring instantaneous vehicle speed on roadways, 2 miles per hour (1.7 knots, 0.89 m/s) [87] compared to 1.8 knots in results presented here. Using X-band marine radar to observe five unique vessels, the tracking algorithm proposed by [33] produced average vessel speeds within 0.69 m/s (1.34 knots) of speeds reported in AIS data, compared to an average error of 0.74 knots in results presented here. Our similar results indicate an effective methodology that can be applied to small vessels that were included in this study.

The specific error bounds estimated in this work are intended to be used as guidance when interpreting tracking information provided by commercial X-band marine radar systems. By including data that were collected at different locations, the results estimate overall error across areas where different types of vessels are active (Figure 4). Some locations were associated with more commercial shipping traffic or conversely more recreational, non-commercial traffic. However, there was more diverse vessel composition at others, including the Point Bonita location in a heavily urbanized area. Different radar hardware (Table 2) and target-tracking systems also aimed to capture various commercial equipment that is commonly used. Therefore, the results provide a comprehensive estimate of error with defined confidence.

Differences in radar-reported data compared to AIS likely arose from established errors associated with radar tracking of target positions. For AIS, SOLAS vessels are required to use GNSS receivers that provide a vessel’s latitude and longitude with high accuracy, usually within 10 m [2]. But target positions identified by radar are limited by quantization that involves converting the range and bearing cell where the target was detected to a latitude and longitude position [28,49,69]. As a result, the positional accuracy is limited by the range and bearing resolution. Radar systems typically have a range accuracy of 1% of the range in use and a bearing accuracy of ±1 degree [27], which is generally less accurate than the positional accuracy of AIS (Table 6).

Change in location over time that is used to estimate SOG and COG could inherit the errors associated with position. Despite the known errors associated with AIS reporting, a higher level of accuracy is required than that of radar target tracking. For example, GNSS receivers used by AIS vessels are required to report SOG within 2% of true speed, or 0.2 knots, whichever is greater [88], compared to the required radar accuracy of 0.5 knots required in testing scenarios. The GNSS receiver accuracy values are required in normal operating conditions which allow for a defined level of rolling and pitching by the vessel at the water’s surface [88]. We expect that the error from this type of motion in AIS data was minimal and falls within the required standard values. Modern satellite-positioning systems use extensive model-based filtering to reduce positional and derivative errors due to inherent uncertainty in both the systems themselves and common noise sources applicable to vessels, such as waves [89]. Due to this extensive body of work and demonstrated performance in real-world conditions, the standards for AIS (and many other operational components) commonly require 95% confidence that measurements will be within limits except under exceptional environmental conditions. Conditions that pushed vessels in our study beyond the limits could have resulted in AIS-reported values with greater error than expected and influenced comparison with radar-reported values, but we expect a relatively low occurrence in the large dataset.

The range and bearing resolution of radar systems are a function of the horizontal beamwidth of the radar antenna which inherently limits the positional accuracy of radar detections. For this reason, the extent to which boaters can rely on radar for collision avoidance has been well studied. Despite the established positional error of radar, which can be somewhat large relative to small vessels, the range and bearing accuracy values in system specifications provide known bounds around reported positions that radar users can expect. The experimental error between radar and AIS positions reported here (34.34 m) falls within the known positional error of vessels less than five nautical miles from the radar (Table 6), which is the range over which most systems were configured to detect targets. In addition to range and bearing accuracy, it is also important to mention cross-range resolution, which is 10 or 20 m depending on the radar antenna (Table 2). Multiple vessels in close proximity may not be realized as separate targets in target tracking, thereby reporting inaccurate positions for the individual vessels. Finally, small movements of the radar antenna and mast from wind loading and structural vibration could affect the accuracy of reported target locations. We expect that potential displacements would be relatively small in scale compared to the established range and bearing error [90]. Masts were 5 m or less in height (see example in Figure 2). The radars used can sustain wind loading up to 70 knots and meet vibration standards for bridge-mounted equipment [65].

The effects of positional error and filtering could explain the variations in SOG and COG in Figure 6 across radar and AIS detections of the example vessels. Objects at the water’s surface are widely subject to oscillatory motion [91,92], so minor fluctuations in SOG and COG over short time periods are expected and also appear similarly in previous work [30,56]. Radar tracking algorithms could have contributed to the smoother profiles of SOG and COG over time compared to AIS, especially noticeable in the fishing and Class B examples. The small-scale fluctuations in SOG and COG over time likely contributed to instantaneous error. But ultimately, the fluctuations across radar and AIS averaged to similar values.

A direct comparison of the radar and AIS tracks is also limited by the synchronization of radar and AIS measurements [49], including differences in both transmission and processing time, which are typically small [58], and the pairing of detections. The interpolation of AIS detections could have provided points synchronized in time with radar detections but would have introduced additional error. Comparing four interpolation methods, Ref. [50] found error to be between roughly 100–130 m on average, larger than the average distance between AIS and radar points associated in this study (34.34 m). Also, AIS detections before or after significant changes in speed or course were not considered, so consecutive detections included in analysis likely had similar speed and course values. Therefore, we expect that interpolation would not have had a large impact on results. The selection criteria used to match concurrent detections prioritized minimal distance between associated pairs to minimize the impact of mismatched reporting time. In Figure 6, changes in COG were detectable in the AIS data before the radar data, primarily in the cargo ship, fishing vessel, and passenger ferry examples. This was likely due to the GNSS utilized by AIS detecting the changes at the vessel before they were detected by radar system. When vessels maneuver, the smoothing filter used in target tracking may not immediately detect a change in COG as it incorporates new target positions and adjusts [28], which could have resulted in the visible lag of radar-reported values compared to AIS.

Vessel speed and maneuverability also likely impacted error. Increased SOG error of vessels traveling above 20 knots (Figure 9) could result from radar tracking errors associated with gate distance, the area surrounding an established target where the target is likely to be detected at the next radar sweep. When a target rapidly changes bearing in relation to the radar, the gate may not update fast enough or be large enough to immediately find the target [28]. This is especially relevant for vessels in close proximity to the radar where they are also subject to the minimum range of detection, which could cause tracking errors. For slow vessels, increased SOG and COG error can be expected due to minimal changes in position, the basis for calculating these variables. Slower vessels may also be maneuvering more frequently, a scenario when tracking accuracy is reduced [28,49,58], also apparent in results presented here (Figure 9). The correlation of error with these variables was somewhat weak although statistically significant (Table 5), suggesting that additional, potentially conflating factors likely impacted error.

The speed at which vessels travel and their maneuverability are inherently associated with their activity and size. For example, fishing activities and gears have been associated with complex movement patterns (e.g., [93]). This high maneuverability is possible due to a smaller size; all fishing vessels in this analysis were 100 m in length or smaller with an average length of 22 m. Thus, the atypically high SOG and COG error of fishing vessels (Figure 8) may be due to frequent maneuvering. This may also be the case for tug, tow, and pilot vessels as they regularly maneuver while assisting ships and are smaller in size (Table 4). Some Class B vessels, which were assumed to be 20 m in length or less, were also associated with high maneuverability (Figure 5). But the 95th percentile of both instantaneous SOG and COG error for Class B vessels was not atypically high, and there were very few outliers. This could be due to the diverse behaviors of non-commercial vessels, i.e., recreational boaters may be simply transiting or engaged in other activities, such as fishing.

In general, most vessel types were associated with instantaneous error near the overall 95th percentile value, but there were some outliers (Figure 8). Outliers may be due to random tracking errors which can occur with radar systems [28], and there were generally less outliers when using track averages. One exception of note was military and law-enforcement vessels; there were more tracks with extreme average error values compared to instantaneous values. This may be due to the high number of associated detections per track for these vessels (Table 4), increasing the chance for random errors that could have been included in averages but missed when randomly selecting detections to evaluate instantaneous error.

There were also noticeable COG error outliers associated with vessels farther than 15 km away from the radar. Most of these vessels with high error were less than 100 m in length, so they were likely tracked near the maximum range of detection for their size. According to Equation (6), vessels farther than 15 km from the radar systems would not typically be tracked if they had less than 3 m air draft. Inconsistent tracking at the edge of the detection range could have contributed to COG error. These vessels had relatively low SOG error, which could indicate that target-tracking algorithms perform better predicting speed than course at large distances, potentially due to the effects of quantization at varying radar ranges. Due to the potential for random radar tracking errors and some elevated errors associated with specific vessel types, it would be prudent to capture multiple data points from vessels within their expected range of detection when monitoring in the field.

Radar can be a valuable primary or secondary data source on a vessel’s activity, but it is especially valuable for vessels that do not participate in any tracking technologies. The design of this study required mostly complete track records of unique vessels from both radar and AIS, but in general, smaller, non-SOLAS vessels usually do not participate in AIS [15,60,94]. Further, larger vessels may turn off AIS, which can be done to purposefully evade detection (e.g., [95]). The intent of this study was to provide error bounds around radar-reported data in the case that AIS data are missing. And the instantaneous error estimate is intended to be used if data from radar itself may be incomplete. Vessel management systems often integrate multiple data sources, including radar, to gather as much information on vessels as possible [20,96]. Since complementary data on smaller vessels may not be available from other tracking systems, it is important that the accuracy of radar data be well understood.

**Figure 10:**
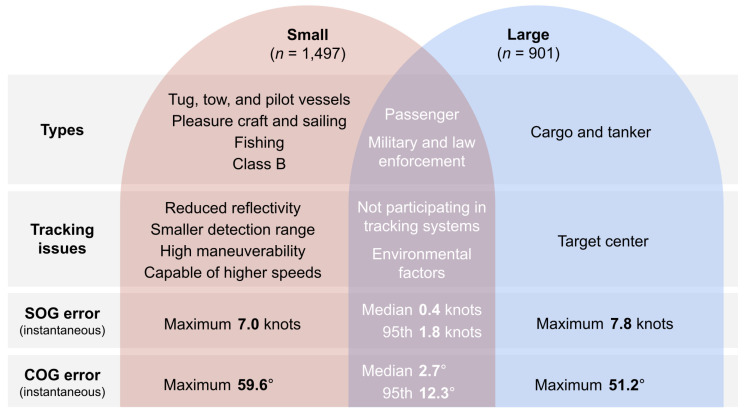
Comparison of small vessels (classified as those less than or equal to 100 m in length) and large vessels (classified as those greater than 100 m in length), including summary statistics (median, 95th percentile, and maximum) for speed over ground (SOG) and course over ground (COG).

This work did not evaluate the overall effectiveness of using radar to detect small vessels, but the potential issues with using radar to monitor these vessels are worth noting. As previously discussed, the range of detection is smaller than that of larger vessels. And with a relatively small RCS, when small vessels are oriented with a parallel aspect to the radar’s line of sight, their RCS becomes even smaller which could thus result in inconsistent tracking. Despite these issues, the radar systems successfully tracked many small vessels, including 747 vessel tracks from Class B AIS (24% of the full dataset) and at least 1497 that were of vessels 100 m in length or less (62% of vessel tracks for which vessel length was known). Further, SOG and COG error was similar across all-sized vessels, and SOG error did not vary by distance from the radar, a variable related to vessel size. Figure 10 provides a comparison of small and large vessels, which demonstrates minimal differences in SOG and COG error.

Despite increased error under some conditions, managers can identify radar-reported SOG and COG accuracy with 95% confidence using the overall instantaneous error estimates of 1.8 knots and 12.4 degrees, respectively, after one minute of radar tracking. Modern radar systems typically have higher tracking accuracy than is required by the SOLAS standards [97], and the error associated with the majority of vessel tracks used in analysis did indeed fall below the standard values. But relying on the 95th percentile error values (roughly three times the standard values) helps ensure that potential sources of error in real conditions are accounted for and that the absolute error is very likely to fall within the estimated range. For example, when enforcing speed limits, such as the 10 knot limit for certain vessels in North Atlantic right whale seasonal management areas in the USA, vessels traveling with radar-reported SOG greater than 11.8 knots could be identified as potential violations of the limit with 95% confidence.

In addition to measuring compliance with speed reduction measures, vessel data also inform risk assessments of whale–ship strikes. At present, vessel speed reduction measures are not widely implemented in high risk areas, and expanding coverage to include these areas would help better conserve whale populations [98]. Speeds reported in AIS data have been used widely to inform risk assessments [99,100,101,102,103]. Radar would be a useful tool for providing data on speeds of smaller vessels that do not transmit AIS for relevant assessments (e.g., [14]). Given that radar is not as well established as AIS for this application, the error bounds provided by this study will allow researchers to estimate uncertainty in radar-reported data on speed, ultimately informing and supporting the interpretation of assessment results. Since smaller vessels made up a large portion of the data analyzed and were not associated with increased error, error bounds can be reliably applied to these vessels in addition to larger vessels. Therefore, radar provides an effective method for measuring the speed of both large and small vessels to inform whale ship-strike mitigation strategies.

## 5. Conclusions

Better understanding and mitigating the risk of strikes between smaller vessels and whales can help support conservation efforts for endangered and threatened species. As tools for monitoring and managing vessel activity beyond AIS are rapidly advancing, marine radar is a practical and approachable sensor system for collecting data on the activity of these vessels. Radar is a traditional tool for marine navigation and is also widely available commercially, which makes it accessible to a large audience, such as enforcement agencies, marine resource managers, and researchers. But with the proprietary target-tracking methods becoming more complex, it is essential that these radar users understand the realistic accuracy, especially for enforcement or scientific applications. This work provides an empirical measurement of system error using a robust data set and statistical approach to be used by those who monitor and enforce regulations in real-time and those who analyze vessel activity to inform management decisions. Vessel speed reduction measures lower the risk of lethal strikes to whales, and measuring the speed of smaller vessels with radar ensures that these vessel types can be included in mitigation strategies. Further, knowing the accuracy of speed measurements supports more reliable reporting and scientific analyses of radar-reported data, so that vessel speed reduction measures can be effectively designed, monitored, and enforced. 

## Figures and Tables

**Table 1:** Time period of data collection.

Location	Start Date	End Date
A	1 August 2023	10 August 2023
B	1 April 2023	5 June 2023
C	1 November 2022	5 July 2023
D	1 April 2022	30 May 2023
E	15 September 2022	30 July 2023
F	1 June 2021	30 May 2022
G	15 April 2022	30 July 2023
H	15 January 2023	30 March 2023
I	1 December 2020	15 August 2021
Point Bonita	1 March 2023	15 May 2023

**Table 2:** Specifications of Furuno radar antennas used [65].

	DRS4D-NXT (Solid-State)	DRS25A X-Class (Magnetron)	DRS25A-NXT (Solid-State)
Minimum range	20 m	25 m	10 m
Range resolution	20 m	20 m	10 m
Bearing resolution	3.9 degrees	1.4–2.3 degrees *	1.35–2.3 degrees *
Radial velocity resolution	1 knot	Not applicable	1 knot
Range accuracy	1% of range in use	1% of range in use	1% of range in use
Bearing accuracy	±1 degree	±1 degree	±1 degree
Rotation speed	24 rotations/minute	24 rotations/minute	24 rotations/minute
Locations	A, B, C	Point Bonita, D, E, F	G, H, I

* Depending on antenna length.

**Table 4:** Average (±standard deviation) length, range, speed over ground (SOG), and maneuverability for the most common vessel types detected. Vessels that did not report dimensions in Automatic Identification System (AIS) data were not included when averaging length. Range, SOG, and maneuverability were averaged across all AIS associated detections.

Vessel Type	Track Count	Associated Detections per Track	Length (m)	Range (km)	SOG (Knots)	Maneuverability (Degrees)
Cargo and tanker	882	18 ± 31	217 ± 65	8.1 ± 5.5	12.3 ± 3.2	1.9 ± 3.4
Military and law enforcement	139	48 ± 115	159 ± 63	12.8 ± 4.6	10.6 ± 2.7	7.3 ± 14.7
Pleasure craft and sailing	142	50 ± 52	56 ± 22	7.9 ± 3.1	11.6 ± 2.8	2.4 ± 5.1
Passenger	274	44 ± 54	55 ± 82	7.0 ± 3.0	11.5 ± 4.8	7.7 ± 13.2
Tug, tow, and pilot vessels	503	21 ± 23	28 ± 13	5.6 ± 3.3	11.6 ± 7.2	13.2 ± 29.2
Fishing	41	17 ± 18	22 ± 9	6.6 ± 4.5	8.4 ± 1.5	6.2 ± 14.1
Class B	747	21 ± 19	-	5.5 ± 2.2	8.8 ± 3.9	7.0 ± 11.6

**Table 5:** Correlation of instantaneous speed over ground (SOG) and course over ground (COG) error with vessel length, range, SOG, and maneuverability.

	Variable	Degrees of Freedom	Correlation Coefficient (ρ)	Significance (*p*)
**SOG error**	Length	2996	−0.028	0.1197
	Range	3095	0.000	0.9828
	SOG	3095	0.058	<0.01
	Maneuverability	3095	0.164	<0.001
**COG error**	Length	2996	−0.022	0.2181
	Range	3095	−0.064	<0.001
	SOG	3095	−0.113	<0.001
	Maneuverability	3095	0.255	<0.001

**Table 6:** Range and bearing error values at example range scales according to the radar specifications identified in Table 2. Approximate bearing-error distance expressed as the arc length of reported bearing error (±1 degree) at given ranges.

Range Scale (Nautical Miles)	Range Error (m)	Bearing Error (m)
0.25	4.63	8.08
0.5	9.26	16.16
0.75	13.89	24.24
1.5	27.78	48.49
3	55.56	96.67
6	111.12	193.94
12	222.24	387.88
24	444.48	775.76

## Data Availability

All data presented in this study are not publicly available as there are safety and security concerns related to openly sharing underlying data that could reveal the exact location of monitoring sites. We confirm that anonymized data can be made freely available to other researchers upon request.
